# Miniaturization of a colorimetric cellulase activity assay on a microfluidic sensor platform

**DOI:** 10.1039/d5ra07446k

**Published:** 2025-11-11

**Authors:** Elisabeth Hengge, Pakapreud Khumwan, Veronica Mora-Sanz, Alvaro J. Conde, Conor O'Sullivan, Andoni Rodriguez, Caroline Hennigs, Martin Smolka, Nerea Briz, Bernd Nidetzky

**Affiliations:** a Graz University of Technology, Institute of Biotechnology and Biochemical Engineering Petersgasse 10-12 8010 Graz Austria bernd.nidetzky@tugraz.at; b Joanneum Research Forschungsgesellschaft – Materials Franz-Pichler-Straße 30 8160 Weiz Austria; c TECNALIA, Basque Research and Technology Alliance (BRTA) Mikeletegi Pasealekua 2 20009 Donostia-San Sebastián Spain; d Micronit BV Colosseum 15 Enschede 7521 PV The Netherlands; e Inmold A/S Teglbuen 10 2990 Nivå Denmark; f Bionic Surface Technologies GmbH Liebenauer Hauptstraße 2-6 8041 Graz Austria; g Naturstoff-Technik GmbH Marie-Curie-Straße 11 27711 Osterholz-Scharmbeck Germany

## Abstract

Enzymes are important commercial products with widespread uses as industrial catalysts, processing aids and analytical reagents. Biological activity is the major critical quality attribute monitored during the enzyme production. Assaying the enzyme activity often requires laborious procedures that are challenging to implement on microfluidic chips for facile on-site use in hand-held mobile devices. Here, we show the integration of a colorimetric assay of cellulase activity into a prototype microfluidic sensor platform on cyclic olefin copolymer foil obtained by roll-to-roll processing. The activity assay was based on resorufin dye release from the corresponding β-cellobioside substrate. Low water solubility of the substrate necessitated a concentration co-optimization for substrate and dimethylsulfoxide co-solvent used in the assay, considering that the co-solvent caused substantial activity loss of the enzyme. A flexible, chip-amenable read-out method was developed based on CIELAB colour space image analysis to quantify both dye release for enzyme activity determination and substrate solubilization at the same time. Information on the substrate solubilization kinetics on chip was crucial to establish the activity assay on the microfluidic platform designed to contain the substrate as a spotted array of resorufin β-cellobioside droplets. We compared the assay conducted in the microfluidic chip with that in conventional microwell plate and found that both formats of analytical measurement could be used with the colour-based read-out method. Overall, we demonstrate cellulase activity monitoring on chip based on identification and optimization of critical parameters of microfluidic assay integration. The analytical approach shown for cellulase may be broadly applicable to the assay of enzyme activity.

## Abstract 2

A novel CIELAB colour space image analysis on microfluidic chips enables cellulase activity and substrate solubility measurement using a colorimetric assay.

## Graphical abstract

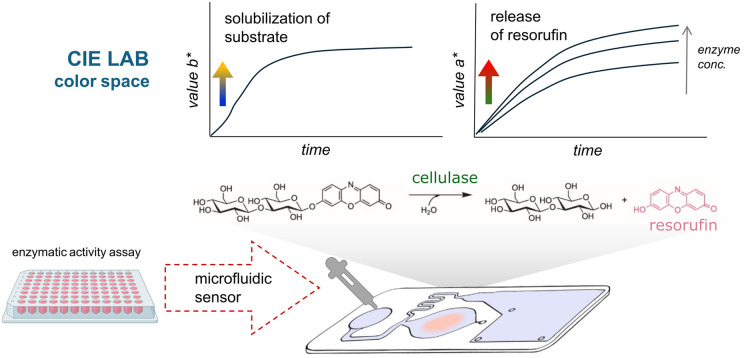

## Introduction

Industrial enzymes have important applications as processing aids in various commercial sectors, including food/feed, detergents/laundry and textiles.^1–3^ They are finding increasing uses as catalysts in organic synthesis and thus contribute to the ongoing transition of the chemical and pharmaceutical industries toward sustainability.^4,5^ Enzymes are manufactured in so-called “fermentations”, which are controlled bioreactor cultivations of microbes that produce the desired enzyme during their growth. Fermentation efficiency is evaluated by the space-time yield of active enzyme in the unit volume of bioreactor culture.^6–8^ The enzyme is usually monitored by its activity, which is measured in dependence of the fermentation time using a dedicated assay.^9^ The single main goal of the activity assay is to quantify the amount of active enzyme through a suitable biochemical reaction performed under well-standardized conditions. Note that the activity assay is not designed in general to assess the enzyme performance in the industrial application considered. Important criterion of the assay is, however, specific measurement of the relevant activity in the complex matrix of sample that fermentation mixtures usually represent. Enzyme assays often involve elaborate procedures that require trained personnel and dedicated facilities for their execution. Transfer of the activity assay onto a multifunctional chip capable of integrating the serial steps of the lab analysis into an all-in-one analytical procedure is promising to advance existing enzyme assays through improved standardization and enhanced automation.^10,11^ These miniaturized devices employ microfluidic channels and compartments to integrate fluid handling in the nano to microliter range, reaction and detection in a single chip. Detection methods commonly include electrochemical, fluorescent or colorimetric read-out.^12–14^ The current study was concerned with the demonstration of a colorimetric cellulase assay on a microfluidic sensor platform. Cellulases are important industrial enzymes with numerous applications in different sectors such as detergent, textile and biofuels.^2,15^ Measurement of their activity is critical not only to monitor the enzyme production by fermentation but also when formulating them into liquid or dried powder products.^10,16^ Colorimetric assays require simple and low cost equipment, do not generally require numerous or expensive reagents and can be scaled down to small volumes making them perfectly suited for portable microfluidic sensor devices.^17,18^

The development of a colorimetric enzyme assay on chip should consider important analogies with enzyme-based biosensors on chip.^19–24^ The enzyme assay on chip differs in that substrate must be provided as reagent. Importantly, in metabolite analysis the enzyme is present in substantial excess and the read-out is usually not affected by the degree in which the total amount of enzyme (spotted, immobilized) is made available.^25–27^ In contrast, enzyme assays require the substrate to be supplied as a reagent, making the substrate concentration within the chip a critical parameter. Inadequate substrate levels can lead to under- or overestimation of enzyme activity, compromising assay accuracy. Therefore, precise control over both the spatial distribution and concentration of the substrate in the miniaturized reaction chamber is essential.^28^ This can be effectively achieved using inkjet printing or spotting techniques.^29,30^ In piezoelectric spotting, defined droplets are generated in the pL to nL range. Importantly, assays cannot be directly transferred from standard laboratory formats, such as microplates, to microfluidic platforms without adaptation. The confined fluid dynamics and reduced optical path lengths in microfluidic chambers influence both reaction kinetics and the signal intensity.^31,32^ Furthermore, successful inkjet printing of reagents is only possible when ink viscosity, homogeneity and surface tension are properly optimized. In addition to technical considerations, cost-efficiency and reagent consumption must also be addressed during assay optimization when targeting industrial applications.

To perform comprehensive analysis of the assay, time-resolved monitoring of the reaction chamber is indispensable. This can be accomplished through image-based colorimetric analysis. These methods do not require specialized equipment^33–35^ and provide complementary information that conventional methods, such as spectroscopy, are missing.^36^ But most importantly, image analysis is less prone to errors, especially when inhomogeneities occur in the reaction chambers. In literature, true-colour sensor platforms for (bio-) sensing of various biomarkers, pesticides or heavy metals for medical, environmental or food safety applications have been demonstrated in recent publications.^33,37–40^ However, colorimetric image analysis for time-resolved assay characterization has not yet been exploited to its full potential.^41–43^ For instance, there are hardly any studies that look at the relative change of value a* compared to b* (in the CIELAB colour space) or at image statistics to gain additional information about the ongoing reactions.

Here, we established an image-based analysis to analyse enzyme assay kinetics based on the CIELAB colour space.^44^ We apply this methodology to successfully implement a colorimetric cellulase activity assay on a low-cost disposable microfluidic device which was previously developed in Mora-Sanz *et al.*^45^ The platform is optimized for application in industrial settings and enables rapid and facile colorimetric measurements. The single use chip is produced by roll-to-roll extrusion coating of cyclic olefin copolymer (COC). The microfluidic design is tailored to ensure a metered volume in the reaction chamber. It enables light to pass vertically to allow optical read-out. To meet the requirements of on-chip applications, the employed cellulase activity assay must be continuous, colorimetric, fully soluble, and free from toxic or hazardous reagents. Based on these criteria, a resorufin-based assay was selected. Originally introduced by Coleman *et al.*^46^ as a fluorescence-based method utilizing resorufin-cellobioside (ReC) as the substrate, the assay exploits the enzymatic cleavage of ReC to release resorufin. Since resorufin exhibits a distinct absorption maximum at 570 nm, the method can be adapted for colorimetric detection. Through image analysis, we were able to monitor the biocatalytic reaction and identify critical parameters for assay integration in the microfluidic platform. Overall, the analytical approach can be applied to numerous other assays and thereby significantly facilitate and streamline assay optimizations.

## Experimental

### Chemicals

Triton X-100, dimethyl sulfoxide (DMSO) and other chemicals were from Carl Roth. *Aspergillus niger* cellulase was from Sigma. The enzyme is a solid preparation and was used as received from the commercial supplier. Resorufin-β-cellobioside (ReC) was from Abcam. Milli-Q water was used. Unless mentioned otherwise, a 0.50 mol per L sodium acetate buffer (pH 6.0) was used in all experiments. ReC was first dissolved in DMSO and then diluted in acetate buffer. ReC solutions contained 5% DMSO (by volume) if not stated otherwise.

### Cellulase activity in microwell plate format

Reactions were carried out in 96 well microwell plates (total volume 50 μL) at room temperature (∼22 °C). Enzyme solution was prepared from the commercial cellulase preparation by mass. Stock solution (50 mg mL^−1^) was diluted into acetate buffer to a concentration in the range 0.01–6.25 mg mL^−1^. The DMSO concentration varied between 0 and 10 vol%, that of ReC between 0.05 and 0.50 mM. A FLUOstar Omega microplate reader (BMG Labtech) was employed for photometric analyses. Optical density (OD) was recorded at 570 nm over a period of 40 min. For data analysis, the linear range between 5 and 20 min was selected, and a linear fit was applied to determine the reaction rate (ΔOD/Δ*t*). Calibration of OD to the resorufin concentration is shown in Fig. S1 of the SI. The slope factor of 4.33 mM^−1^ was obtained with the set-up used. The reported molar extinction coefficient at 570 nm is 58 500 M^−1^ cm^−1^. The DMSO concentration was without effect on the resorufin OD within the calibrated range (0.001–0.100 mM), as shown in Fig. S1. The observed ΔOD/Δ*t* was converted into a molar rate in μmol (L^−1^ min^−1^) using the calibrated slope factor. The standard activity assay of the cellulases used 0.5 mM ReC and 5 vol% DMSO. The commercial preparation was determined to have a specific activity of 0.34 ± 0.05 U g^−1^ (*N* = 6). One unit (U) is the enzyme amount that releases 1 μmol resorufin/min under the conditions used.

### Spotting of the ReC substrate

Stock solution (10 mM) of ReC was prepared in acetate buffer containing 5 vol% DMSO and diluted to the desired concentration. Dispensing was carried out using a sciFLEXARRAYER S12 spotter (Scienion AG, Berlin, Germany) equipped with an uncoated PDC90 nozzle. Spotting was performed at room temperature (∼22 °C) and at 60% humidity. The total volume spotted in microwell plates was 2.5 μL, that spotted in microfluidic chips was 0.6 μL. Rectangular layouts (5 × 5, 10 × 10, 12 × 12) were used as microarrays. The corresponding volume/spot was 24.0, 6.00 and 4.17 nL. The voltage and the pulse of the piezo-dispensing capillary were optimized individually for each liquid formulation and the droplet formation was checked before each run *via* the built-in camera. After spotting, the plates and microfluidic chips were placed directly in a closed compartment with silica gel until the substrate was completely dried. They were sealed afterwards and stored at 4 °C until further use.

### Cellulase assay in microfluidic chip format

**Fig. 1:**
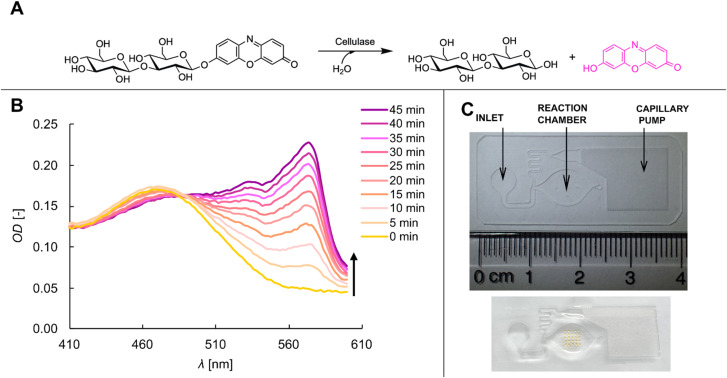
Assay used for the colorimetric detection of cellulase activity on microfluidic chip. (A) Reaction scheme showing hydrolysis of ReC substrate by the cellulase to release the 570 nm chromophore resorufin. (B) Temporal evolution of the absorption spectrum of the reaction mixture upon conversion of ReC by the cellulase. The stacked absorption spectra are coloured by reaction time, that is, yellow for *t* = 0 min and violet for *t* = 45 min, indicated by the black arrow. Reaction conditions: 0.1 mM ReC, 5.0 mg mL^−1^ cellulase in acetate buffer containing 5 vol% DMSO. (C) Top: photograph of the microfluidic chip fabricated from COC foil showing the inlet, where the enzyme containing sample is applied, the reaction chamber and the capillary pump. Bottom: photograph of ReC substrate spotted in a 5 × 5 microarray in the reaction chamber.

The microfluidic chip used is shown in Fig. 1C. Its functional microstructure is fabricated from an extrusion-coated COC foil. The total volume of the chip is 12 μL. Spotting of ReC was done in the reaction chamber area of the chip (Fig. 1C) using the methods described above. For analytical use, 10 μL of hydrophilic liquid were applied for surface coating on the micropillars of the capillary pump. Note that COC is a hydrophobic material and so hydrophilic coating is critical for capillary pump function. The chips were then closed with a laser-cut pressure sensitive adhesive tape. Analyte (50 μL) of varied cellulase concentration was applied to the inlet chamber. The cellulase sample was diluted in acetate buffer containing 0.1 vol% Triton X-100 and 5 vol% DMSO. Image analysis was used to evaluate the dynamic colour formation in the chip.

### Colorimetric image analysis

Images were recorded during the enzymatic reaction for up to 60 min. A Huawei P20 EML-L29 smartphone equipped with an EMUI 12.0.0 (Android based) operating system was used to record the videos. Microwell plates or microfluidic chips were placed in a white Styrofoam box and illuminated by a uniform white light from top. The smartphone was placed next to the light, which ensured constant position of the camera lens relative to the reaction volume. Image analysis was performed in MATLAB. First, a white region was selected for normalization. Then, the relevant region of interest (central area of the reaction chamber) was cropped by the operator and the mean values of the red, green and blue channels were calculated. The channels were normalized by the mean grey value (for white balancing), excluding saturated pixels and subsequently converted to the CIELAB colour space *via* the MATLAB function *rgb2lab*.^47^ The mean and standard deviation of the L*, a*, and b* values within the region of interest were calculated for each image, providing a quantitative representation of colour characteristics. Each region of interest contained roughly 100 × 100 pixels. A schematic representation of the experimental setup and steps conducted for image analysis is presented in Fig. S2 (SI).

## Results and discussion

### Integration of cellulase assay into the microfluidic system

The term cellulase stands for a mixture of glycoside hydrolases that depolymerize cellulose chains.^16,48^ Cellulose is insoluble and its hydrolysis releases products unsuitable for the direct detection by absorbance. The assay based on resorufin release from ReC (see Fig. 1A) offers the advantage of sensitive colorimetric detection, yet it also comes with limitations that are mentioned briefly. ReC is a completely artificial substrate for the cellulase ensemble of enzymes.^46^ It lacks the central feature of solid material that the cellulose represents. Unlike cellulose which requires the synergistic activity of endo- and exo-cleaving enzymes for efficient degradation,^16,49^ a short-chain substrate such as ReC is hydrolysed by just one or maximally two event of glycosidic bond cleavage. Synergy among the different enzymes is absent during ReC hydrolysis. The ReC-based activity assay is useful to monitor the enzyme amount released during the production, provided that the composition of the enzyme mixture does not change significantly in the process.^48^ The ReC assay is not designed to show the immediate cellulase activity with cellulose. Referencing is required to establish the quantitative relationship between activity with ReC and target substrates in technological applications.

Assay integration into the microfluidic platform necessitates facile and sensitive detection by absorbance and solid substrate cannot be used. The release of resorufin fulfilled the requirements of detection (see Fig. 1B) and ReC is a soluble substrate. Challenges of ReC solubilization are discussed below. The molar extinction coefficient (*ε*) of resorufin is ∼58 mM^−1^ cm^−1^ (570 nm) at the pH of the enzymatic reaction. It is worth noting that 4-nitro-phenol which is often used as chromogenic group in assays of hydrolase enzymes exhibits low absorbance (*ε* = 0.2 mM^−1^ cm^−1^; 405 nm) at pH ≤ 5.5.^50^ Conversion into the more strongly absorbing 4-nitro-phenolate anion requires change to alkaline pH conditions (pH ≥ 7.5) that are not well compatible with the cellulase activity. Conventional enzyme assays therefore use pH-shift to ≥8 after the enzymatic reaction to enhance the detection. For assay on chip, however, a one-step procedure with continuous detection is desired. High signal intensity per unit volume is important due to the limited optical path length of approximately 200 μm. The requirements were met by resorufin release (Fig. 1B).

The microfluidic architecture of the sensor chip is shown in Fig. 1C. The filling of the microfluidic channels is driven by the capillary pump based on micropillars. When the reaction chamber is filled completely, the capillary stop valve at the end prevents further flow. The excess liquid in the channel is removed by the capillary pump, creating a compartmentalized and metered volume in the reaction chamber of 12 μL. The COC, in which the microfluidics are imprinted, as well as the adhesive tape to cover the chip exhibit high transparency in the visible spectrum, allowing light to pass through vertically. This design not only permits absorbance-based detection of the enzymatic reactions but also supports both visual and image-based monitoring of colorimetric changes. Reagents are combined during chamber loading without subsequent manipulation, ensuring a facile and automatable operation of the assay. In the following, the applicability of the ReC assay is first analysed in microwell plates and then transferred to the microfluidic chip.

### Microwell plate cellulase assay

**Fig. 2:**
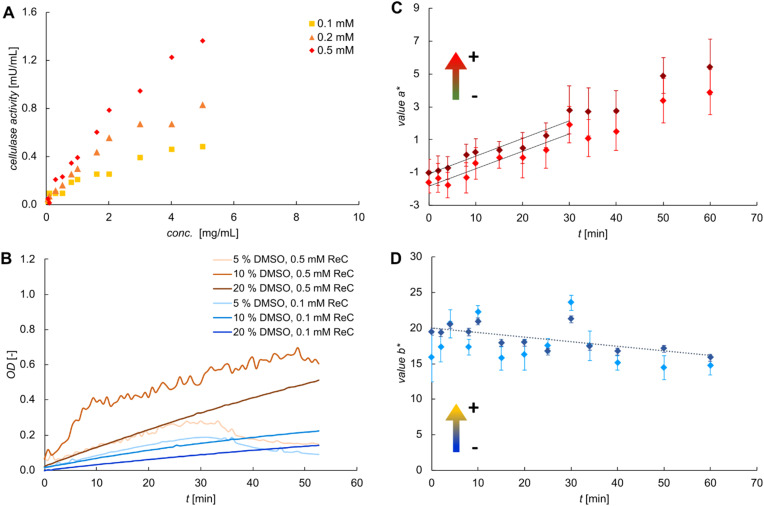
Assay of cellulase concentration by activity in microwell plates and development of image analysis-based method of data evaluation. (A) Dependence of the volumetric reaction rate on the concentration of cellulase for reactions done at different concentrations of ReC substrate. Inset: representative time courses of the OD for enzymatic reactions performed at 0.1 mM ReC. (B) Temporal evolution of OD under conditions of varied concentrations of ReC and DMSO for reactions performed with 5.0 mg mL^−1^ cellulase. (C and D) Image analysis of the data showing value a* (C) and b* (D) for reaction performed with 0.1 mM ReC, 5 vol% DMSO and 1.0 mg mL^−1^ cellulase. Results of two separate measurements are shown. Straight-line fits of the data are shown in panel (C), with slopes of 0.105 (*R*^2^ = 0.89; red) and 0.108 (*R*^2^ = 0.91; dark red) agreeing well with one another. Straight-line fit in panel (D) with slope −0.065 (*R*^2^ = 0.5).

Resorufin is well-known for its use as fluorophore or chromophore in analytical applications.^51–53^ For cellulase activity determination, ReC has been used so far only as a fluorogenic substrate.^46^ Hence, to test ReC in a photometric setting of measurement of chromophore release, we initially characterized the absorption behaviour of the ReC-resorufin system in microwell plates using soluble reagents. Fig. 1A shows the change in absorption spectrum for a reaction mixture (0.1 mM ReC) with cellulase. Fig. S3-A (SI) additionally shows a visual representation of the colour changes after the enzymatic reaction. The OD as a function of time is exemplarily shown for the 0.1 mM ReC reaction in the inset of Fig. 2A. The time courses were linear for 10–30 min, depending on the enzyme concentration used, which allowed for convenient determination of the volumetric resorufin rate in μmol (mL^−1^ min^−1^) under all conditions. The relationship between enzymatic rate and enzyme concentration was investigated at different substrate concentrations (Fig. 2A). The assay exhibited a broad detection range for cellulase, from 0.04 to 5.0 mg mL^−1^, with a linear response shown up to ∼2.0 mg mL^−1^. However, the resorufin rate was dependent on the ReC concentration, indicating that the enzyme was not saturated with substrate under the conditions used. Enzyme assays are usually designed to employ saturating substrate concentration. Low solubility of ReC in water prevents the use of concentrations exceeding ∼0.5 mM at 5 vol% DMSO. Regardless of the limitations in the useable ReC concentration, the linear relationship of rate *vs.* enzyme concentration allows for the quantification of cellulase by activity under all conditions used (Fig. 2A). However, we add the cautionary note that, since the assay response is dependent on the ReC concentration (Fig. 2A), it is critical that the enzyme assays are performed under conditions of reproducibly defined substrate concentration. We show below that during assay operation on-chip the solubilization of ReC presents a challenge. Overall, however, the results establish that the resorufin-based assay provides a sensitive method for cellulase quantification by activity.

Next, we examined the influence of DMSO concentration on the assay performance (Fig. 2B). Low ReC concentration (0.1 mM) combined with high DMSO content (10–20 vol%) gave stable and continuous increases of the OD (dark blue traces, Fig. 2B). By contrast, high ReC concentration (0.5 mM) or low DMSO content (5 vol%) resulted in increased signal noise and abrupt fluctuations in the absorbance curves (orange trace, Fig. 2B). Additionally, prolonged incubation (*t* >30 min) led to a gradual decline in the OD (light orange/blue traces). These effects are attributed to the solubility behaviour of ReC and resorufin both of which are only sparingly soluble in water and benefit from stable solubilization in the presence of the organic co-solvent. Moreover, we consider that the ReC substrate is added from stock solution in pure DMSO to the acetate buffer used for the enzymatic reaction. Change in the solvent composition might promote partial aggregation of the ReC, thus leading to signal instability due to enhanced light scattering. Additionally, the effective substrate concentration available to the enzyme may be decreased due to aggregation. However, use of DMSO co-solvent is restricted by the effect of DMSO on cellulase activity, as seen from the results in Fig. 2B. Increase in the DMSO content from the standard of 5 vol% to 10 vol% and 20 vol% resulted in enzyme activity reduced by, respectively, 22% and 62%. It was critical, therefore, that the DMSO usage achieved balance between solubilization and retention of enzyme activity. All subsequent experiments were conducted between 0.1 mM and 0.25 mM ReC in 5 vol% DMSO. Only data recorded in the first 30 min was used for analysis.

### Colour image analysis for evaluation of the enzyme assay

An increasing number of studies shows that colorimetric image analysis represents a practical solution of quantitative read-out in chip-based sensing applications. Having the integration of the cellulase assay on microfluidic chip mind, we sought to develop detection by image analysis as an alternative to single-wavelength spectrophotometric detection. We considered the possible advantage of time-lapse image analysis-based (*i.e.*, effectively visual) monitoring of the reaction to identify processes (*e.g.*, turbidity formation due to aggregation of substrate or product) connected to, or happening while, the enzymatic conversion takes place. Conventional 570 nm detection cannot provide this information. Scanning the full wavelength range (Fig. 1B) requires instrumentation not compatible with use on chip. The CIELAB colour space was identified to be a best-suited colour model for the time course evaluation of ReC hydrolysis. In contrary to other colour representation, such as the RGB (red-green-blue), the CIE colour spaces are linearly proportional to the visual impression.^54,55^ From all CIE colour spaces the LAB system was chosen because a colour change can be described by the relative change of a single value, a* or b* which are independent of the lightness L*.^17^ Hence, the L*, a* and b* coordinates are the first choice to quantify colours and compare them with the visual impression of the naked-eye.^44,56^ The values a* and b* represent opponent colour axes: negative a* values indicate green, positive a* values indicate red, negative b* values indicate blue, and positive b* values indicate yellow. In Fig. 2, the temporal evolutions of values a* (panel C) and b* (panel D) during the conversion of ReC are presented. Value a* exhibited a pronounced increase. Value b* showed a slight decrease. Due to the absorption at 570 nm, the colour impression of the reaction volume changed from yellow to pink during the conversion. This was reflected in the observed increase in value a* (shift towards red) and decrease in value b* (slight shift towards blue). For further quantification of the enzymatic kinetics, value a* was chosen as it exhibited a higher relative change in absolute value and followed the anticipated time course for the release of resorufin. The slope of the linear fit (*k*_a*_) could be directly correlated to that of OD *vs. t* obtained from the spectroscopic analysis shown in Fig. 2A (inset), with a conversion factor of ∼120. This correlation was utilized to calibrate the image-based analysis, thereby providing quantitative data on the enzyme concentration as measured by activity. To illustrate this, in case of 1.0 mg mL^−1^ of applied cellulase, the slope *k*_a*_ (=0.107) corresponds to a reaction rate of 0.21 mU mL^−1^.

The standard deviation within the region of interest is represented as error bars in Fig. 2C and D. It directly reflects the colour uniformity across the reaction chamber. For microplate measurements, the observed deviations are primarily due to reflections and shadowing effects caused by the well walls. However, the colour uniformity is also directly linked to the homogeneity of the reaction volume which will be used later to evaluate the re-solubilization. Reproducibility of the analysis was evaluated by performing the same reaction twice in two separate microwell plates (Fig. 2C). The calculated values for *k*_a*_ deviate from each other by only 3%. These results confirm that the presented method is reliable for the evaluation of enzymatic activity based on colorimetric image analysis and that it provides quantitative information on the reaction kinetics.

Approaches comparable to the one used here can be found in the literature. Baş *et al.*^57^ characterized the conversion of glucose by a glucose oxidase using two different dyes, iodine and quinone. Value b* was identified to be best suited for the analysis of the iodine method, value a* for the quinone method. Demirkol *et al.*^55^ presented a microfluidic device that uses the absolute value of the colour vector 
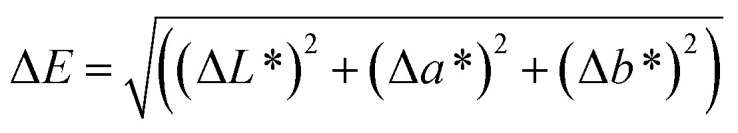
 in the CIELAB colour space for the correlation to the activity of glucose oxidase or laccase. In both assays, the green-coloured radical cation of 2,20-azino-bis(3-ethylbenzthiazoline-6-sulfonic acid) is formed. Δ*E* is commonly used when quantification of the total visual colour impression is desired. In contrast to the earlier studies mentioned, the current work employs the discrete evaluation of colour components a* and b* and hence offers complementary information on solubilization of the substrate (see the next section) and the conversion rate. Additionally, the approach used here is very well-suited for analysis of complex colour changes.

### Utilization of spotted ReC substrate

Design of one-step cellulase assay on microfluidic chip involved integration of the ReC substrate into the reaction chamber as a spotted reagent (Fig. 1C and D) to be dissolved into the assay when the liquid sample is flown in. Spotting of the reagent allows precise control over the volume and position of the deposition and can be incorporated in the roll-to-roll process. In piezoelectric spotting, defined droplets are generated by a pressure wave generated from a suitable voltage pulse. The successful formation of these droplets strongly depends on the surface tension and viscosity of the ejected liquid.^30^ To examine the feasibility of spotting the ReC in principle, we first performed experiments in the microwell plate. The spotting parameters (volume and concentration) and reaction conditions were chosen based on the results obtained in the previous section. Freshly spotted ReC in microwells can be seen in Fig. S1B and C (SI). It is evident that some spots were agglomerated or dragged to the well walls.

**Fig. 3:**
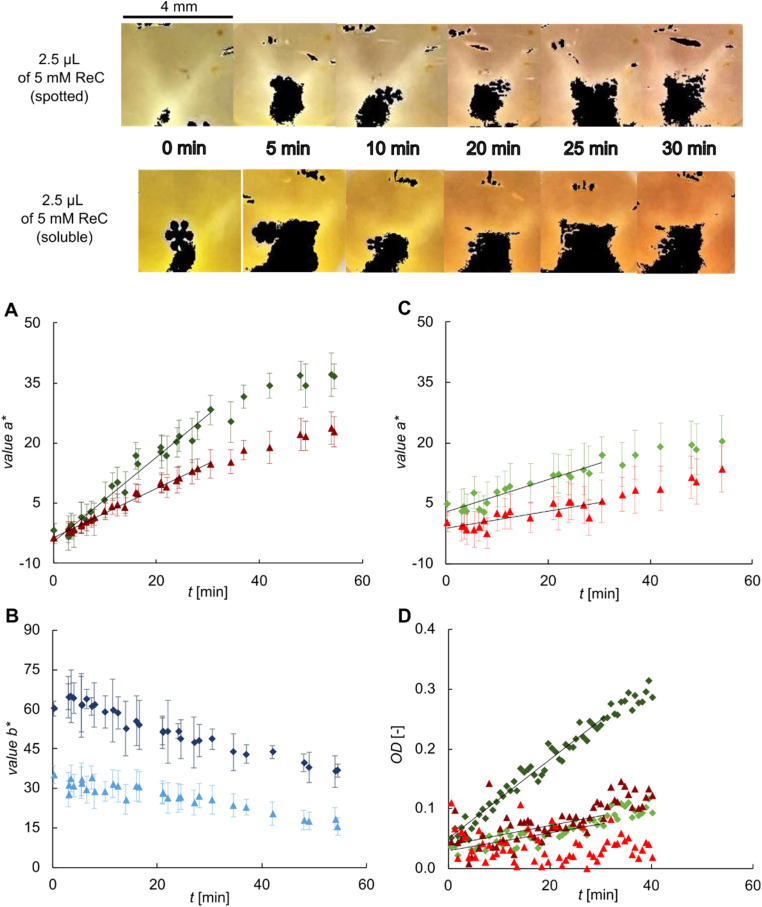
Comparison of cellulase activity assay using soluble and spotted ReC substrate. Top part: selection of images recorded during the conversion of spotted and soluble ReC (indented final concentration: 0.25 mM) using 5.0 mg mL^−1^ cellulase in acetate buffer containing 5 vol% DMSO. Total volume 50 μL, 5 × 5 microarray. Black areas on the image indicate saturated pixel that were excluded from analysis. (A–D) Squares: soluble ReC, triangles: spotted ReC. (A and B) Values a* and b* determined from the image data set presented above. (A) Slope of linear fit: for soluble ReC *k*_a*_ = 1.041 (*R*^2^ = 0.96) and for spotted ReC *k*_a*_ = 0.624 (*R*^2^ = 0.99). (C) Value a* determined for the same reaction condition but applying 1.0 mg mL^−1^ cellulase. Slope of linear fit: for soluble ReC *k*_a*_ = 0.381 (*R*^2^ = 0.86) and for spotted ReC *k*_a*_ = 0.213 (*R*^2^ = 0.62). (D) OD recorded for the same reactions as shown in (A) and (C). Calculated activity: 5.0 mg mL^−1^ cellulase, soluble ReC: 1.52 mU mL^−1^; spotted ReC, 0.37 mU mL^−1^. 1.0 mg mL^−1^ cellulase: soluble ReC, 0.35 mU mL^−1^; spotted ReC: not measurable.

A direct comparison in the enzymatic reaction between soluble and spotted ReC is shown in Fig. 3. Preliminary visual inspection, shown in Fig. 3 top, revealed a diminished colouration of the reaction chamber with spotted ReC, hinting at an incomplete solubilization of the substrate. The suggestion is further supported by the visibility of residual spots in the upper right corner of the images throughout the whole incubation time. Note: the black regions mark pixels that were excluded due to saturation of at least one of the colour channels.

Fig. 3A and B shows the evolution of value a* and b* for reactions with spotted and soluble ReC. Value a* (Fig. 3A) increased under both conditions, as expected, but the enzyme activity determined from the data was decreased by 34% (1.7-fold) when spotted instead of soluble ReC was used. Value b* (Fig. 3B) decreased over time due to increase of the blue colour component. Note: value b* represents the yellow to blue colour axis and hence, it also gives insight into the ReC dissolution process. Firstly, directly at the start of the measurement, the reaction with soluble ReC exhibited two times the value b* of the reaction of the spotted ReC. Secondly, a closer look at the first ∼30 min of incubation shows that the value started to decrease immediately for the soluble ReC reaction, whereas it stayed constant for the spotted ReC reaction. The dynamics of value b* can be explained as follows. Value b* increases as result of the ongoing solubilization of ReC, it decreases on cleavage of ReC with resorufin release. The constant value b* in the reaction of spotted ReC reflects a steady state of the two processes, implying a constant concentration of ReC lower than in the reaction of soluble ReC. Additionally, value a* was also evaluated for the same reaction conditions but applying 1.0 mg mL^−1^ cellulase (Fig. 3C) instead of 5.0 mg mL^−1^. The difference in the measured activity (*k*_a*_) between the soluble and spotted ReC reaction was 36% (1.55-fold) which was quite similar as found before with higher cellulase loading.

As the spotting experiments were performed in microwell plates, spectroscopic analyses were done on the same reactions in parallel (Fig. 3D). The results revealed similar trends. However, the OD exhibited strong fluctuations, most likely caused by diffuse light scattering due to molecular aggregation of ReC. Colorimetric image analysis exhibited significantly reduced data scattering, evidently because it is not sensitive to aggregation phenomena, thus highlighting the benefits of the method for data evaluation.

Overall, these results showed that the solubilization of spotted ReC exerted a substantial influence on the assay performance. We realized that efficient solubilization of the substrate might represent the biggest obstacle in the miniaturization of the enzyme assay. Controlled solubilization of reagents after spotting is rarely discussed in the context of biosensing^30,58^ but it is known as one of the most important aspects in printing pharmaceuticals for controlled release of drugs. Of course, the pharmaceutical application imposes considerably more stringent requirements going beyond the mere solubilization of the substances, but previous studies show that among critical spotting parameters, droplet formation and microarray layout can have a substantial positive influence on solubilization.^29,59^

### On-chip implementation

Spotting parameters from the microwell plate experiments were transferred directly to the microfluidic chip, except that 0.6 μL (instead of 2.5 μL) ReC solution in DMSO were used. Note that 0.1 vol% of Triton X-100 was added to decrease surface tension and increase self-filling rate in the microchannels. Different concentrations and microarray layouts were examined. Value a* and b* were analysed for reactions with cellulase at 1.0, 4.0 and 5.0 mg mL^−1^ concentration.

**Fig. 4:**
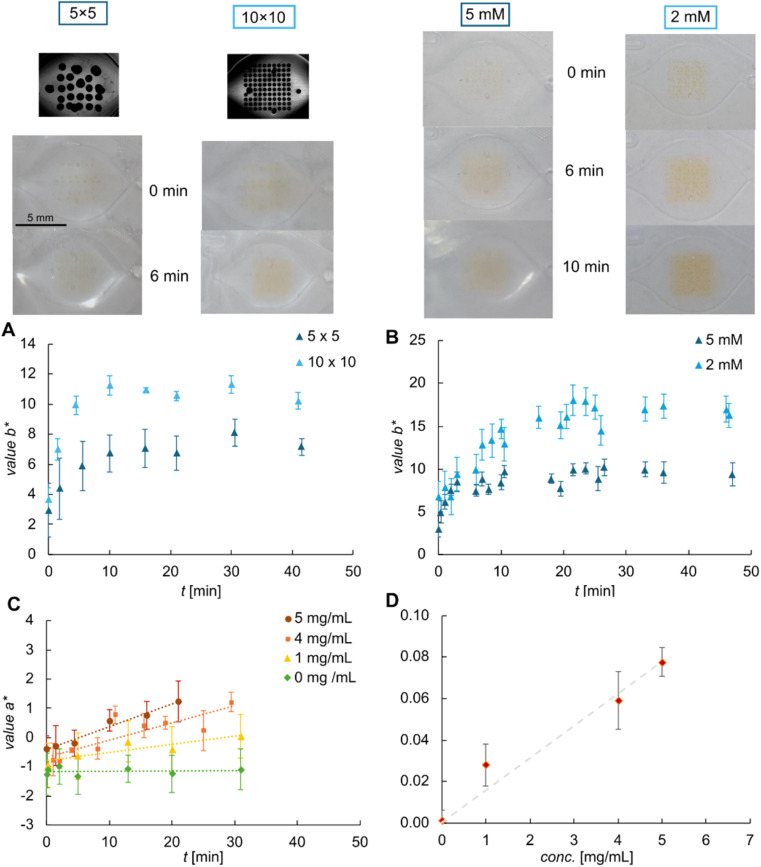
Implementation of the cellulase activity assay on the microfluidic chip. Top: images of the reaction chamber after application of the sample (5.0 mg mL^−1^ cellulase in acetate buffer containing 5 vol% DMSO and 0.1 vol% Triton X-100) for the different microarray layouts (left, 2.0 mM ReC, total volume 0.6 μL) and ReC concentrations used (right, 10 × 10 array, total volume 0.6 μL). The corresponding evolution of value b* is shown in (A) and (B), samples contained 4.0 mg mL^−1^ cellulase. (C) Dependence of value a* on the cellulase concentration. Final concentration of ReC 0.1 mM (10 × 10 microarray, 0.6 μL of 2.0 mM spotted). Results: 0 mg mL^−1^ (*k*_a*_ = 0.001, *R*^2^ = 0.1), 1.0 mg mL^−1^ (*k*_a*_ = 0.028, *R*^2^ = 0.83), 4.0 mg mL^−1^ (*k*_a*_ = 0.059, *R*^2^ = 0.72), 5.0 mg mL^−1^ (*k*_a*_ = 0.078, *R*^2^ = 0.97). (D) *k*_a*_vs. cellulase concentration as determined in (C), error bars represent the standard deviation. Slope of linear fit = 0.015 (*R*^2^ = 0.98).

First, the spot size will be discussed. Two different microarray layouts, 5 × 5 and 10 × 10, were chosen for evaluation and the same activity assay was performed in each case. In Fig. 4, images of the reaction chamber are presented. The image recorded from the in-line camera (top left) shows that agglomeration of spots occurred for the 5 × 5 array. Spot agglomeration is known from earlier works to be detrimental for solubilization.^60^ On images taken after filling the chamber with the reaction mixture there were still residual spots visible, even after 6 min of incubation. In contrast, the spot array at the 10 × 10 layout was well separated with no evidence of agglomeration. Significantly, the reaction chamber appeared homogeneous (no individual spots visible) after a few minutes of incubation. For better resolution of the images, please see the digital version of the manuscript. Fig. 4A shows the concomitant evolution of value b*. Interestingly, the temporal evolution exhibited an increase in b* which appeared to be contradictory to the observations in microwell plates. As already discussed, an increase in b* was interpreted to arise from the ongoing solubilization of ReC (increase of the yellow colour component). Hence, the measured evolution of b* indicated a slower solubilization process compared to the reaction in wells. This result can be well explained by the reduced fluidic movement in the reaction chamber due to the geometrical constraints. Comparing the two microarray layouts, the solubilization seemed to evolve simultaneously but the 10 × 10 array reached a total higher value. As the same total amount of ReC was spotted in each case, this result shows that solubilization was favoured for smaller spot sizes and hence larger microarrays. Of note, the homogeneity of the reaction chamber assessed by the standard deviation nicely reflected these findings. Evolution of value a* (Fig. S4 in SI) exhibited a suppressed increase (*i.e.*, a lag phase) up to 10 min of incubation, being also in-line with delayed solubilization. Additionally, 12 × 12 arrays were tested under the same conditions, but no improvement was observed compared to 10 × 10 arrays (see Fig. S5 in the SI). Thus, a 10 × 10 was identified as the suitable microarray size for the particular application considered within the experimental setup used.

In a next step, the influence of different concentrations of stock solution was evaluated. Images taken for enzymatic reaction using stock solutions of 2.0 and 5.0 mM are shown in Fig. 4 on the top right (10 × 10 microarray). One can already recognize visually that spots remain at the higher concentration, even after 10 min of incubation. It is also visible that the colour change was much more intense at the lower concentration where all individual spots had disappeared after a few minutes of incubation. Both findings suggested that 2.0 mM stock solution was preferable over 5.0 mM. Fig. 4B shows the corresponding evolution of value b* that very well supports these conclusions. The increase of b* was nearly double as high for 2.0 mM as for 5.0 mM.

Summarizing these findings, the best conditions for spotting ReC were identified as 0.6 μL of 2.0 mM spotted in a 10 × 10 array. The response of the microfluidic sensor platform thus obtained was evaluated based on the value of a* and results received upon the application of varying cellulase concentrations are shown in Fig. 4C. The calculated values for *k*_a*_ demonstrate a linear behaviour within the observed range. Consequently, the sensitivity (d*k*_a*_/dconc.) of this image-based methodology can be estimated as 0.015 mg mL^−1^ cellulase. Note, that due to the significantly decreased liquid height in the reaction chamber, here the colour intensity and absolute colour change was strongly reduced compared to the reaction in microwell plates.

Importantly, the results in Fig. 4B reveal that solubilization of ReC continues for up to 20 min of incubation. This time range of solubilization overlaps with the time range used for determination of the enzyme kinetics. Hence, for further application of the assay in bioprocess monitoring, suitable calibration is needed. The *k*_a*_ values for known cellulase concentrations can be correlated to the specific activity determined for the corresponding reaction in microplates (Fig. 2A) which in turn allows for the (semi)-quantitative analysis of cellulase activity in bioprocesses. Building upon this work, the industrial application of the microfluidic system, including (automated) production of the functional chip, integration with a dedicated reader device, validation using industrial samples, and shelf-life evaluation, is the topic of further research (Mora-Sanz *et al.*^45^). Availability of standardized ready-to-use chip in larger number will allow for full statistical assessment of the analytical method on chip.

## Conclusions

One-step colorimetric assay of cellulase activity based on resorufin release from ReC was established and integrated into a microfluidic chip sensor platform (Mora-Sanz *et al.*^45^). Solubility control of the poorly water-soluble ReC substrate was critical for transfer of the assay from the conventional microwell plate format to the advanced format of the functional chip. Image analysis with the CIELAB colour space was developed for convenient and sensitive, chip-integrated reaction monitoring in two essential respects. The value a* was used to follow the enzymatic reaction by resorufin release. The value b* value served to track the solubilization of the spotted ReC substrate. Due to the kinetic properties of the cellulase that cannot be fully saturated with the substrate in the useable range of maximally ∼0.5 mM ReC, the enzyme assays must be performed reproducibly at same conditions of the substrate concentration. Monitoring the rate and the extent of substrate solubilization from the spotted microarray of ReC droplets was possible based on the dynamics of value b* and was crucial for establishing the optimized on-chip operation of the assay. Spotting the substrate in a suitable microarray format was critical for substrate supply on the chip, as revealed by image analysis. An additional advantage of the method of image analysis is its flexible use independent of a dedicated optical reader device. The approach shown here for assaying cellulase on chip could be extended to numerous other industrial hydrolases whose activities are measured with colorimetric substrates of analogous type as ReC.^61–63^ Collectively, this work underscores the potential of colorimetric assays in microfluidic platforms for the characterization of enzymatic reactions. The developed colorimetric image analysis approach provides a powerful tool for enzyme kinetic studies and biosensor optimization, with broad applicability across biotechnological and analytical domains.

## Author contributions

Conceptualization: E. H. and B. N.; methodology and investigation: E. H. and P. K.; visualization: E. H.; resources: C. S., A. R. and C. H. supervision: B. N.; project administration: V. M. S., N. B. and B N.; writing – original draft: E. H. and B. N. writing – review & editing: all authors.

## Conflicts of interest

There are no conflicts to declare.

## Supplementary Material

RA-015-D5RA07446K-s001

## Data Availability

The data supporting this article have been included in the manuscript and as part of the supplementary information (SI). Supplementary information is available. See DOI: https://doi.org/10.1039/d5ra07446k.
