# Supplementary material for: Miniaturization of a colorimetric cellulase activity assay on a microfluidic sensor platform
Source: RSC Adv. 2025 Nov 11;15(51):43866–76. doi: 10.1039/d5ra07446k (PMC12604091; doi:10.1039/d5ra07446k)
Supplement: RA-015-D5RA07446K-s001 [file RA-015-D5RA07446K-s001.pdf]

Supplementary Information (SI) for RSC Advances.
This journal is © The Royal Society of Chemistry 2025

Supplementary Information

Miniaturization of a Colorimetric Cellulase Activity Assay on Microfluidic Sensor Platform

Elisabeth Hengge,[a] Pakapreud Khumwan,[b] Veronica Mora-Sanz,[c] Alvaro J. Conde,[d] Conor O'Sullivan,[e] Andoni Rodriguez,[f] Caroline Hennigs,[g] Martin Smolka,[b] Nerea Briz,[c] and Bernd Nidetzky*[a]

[a] Graz University of Technology, Institute of Biotechnology and Biochemical Engineering, Petersgasse 12, 8010 Graz, Austria, [b] Joanneum Research Forschungsgesellschaft - Materials, Franz-Pichler-Straße 28, 8160 Weiz, Austria, [c] TECNALIA, Basque Research and Technology Alliance (BRTA), Mikeletegi Pasealekua 2, 20009 San Sebastián Spain, [d] Micronit BV, Colosseum 15, Enschede 7521 PV, The Netherlands, [e] Inmold A/S,Teglbuen 10 2990, Nivå, Denmark, [f] Bionic surface technologies GmbH, Liebenauer Hauptstraße 2-6, 8041 Graz, Austria, [g] Naturstoff-Technik GmbH, Marie-Curie-Straße 11, 27711 Osterholz-Scharmbeck, Germany

Supplementary Figures

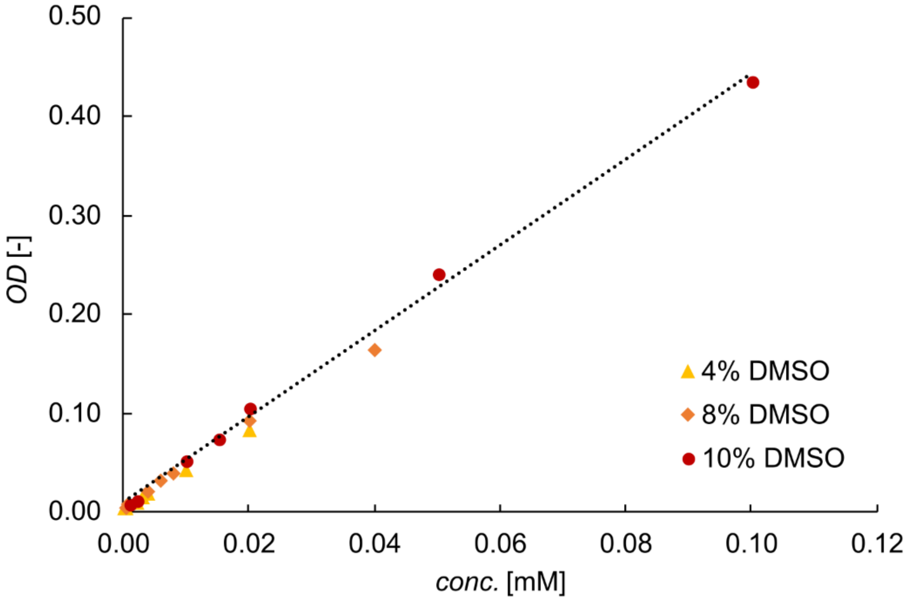

Figure S1. Resorufin calibration curve in acetate buffer for determination of the molar absorbance. To determine any influences of DMSO, different concentrations were applied. Red (cycles): 10 vol%, orange (diamonds): 8 vol%, yellow (triangles): 4 vol%. Reaction conditions: in acetate buffer, 50 µL total volume, RT. Slope of linear fit = 4.33 mM^{-1} (R^2 = 0.997).

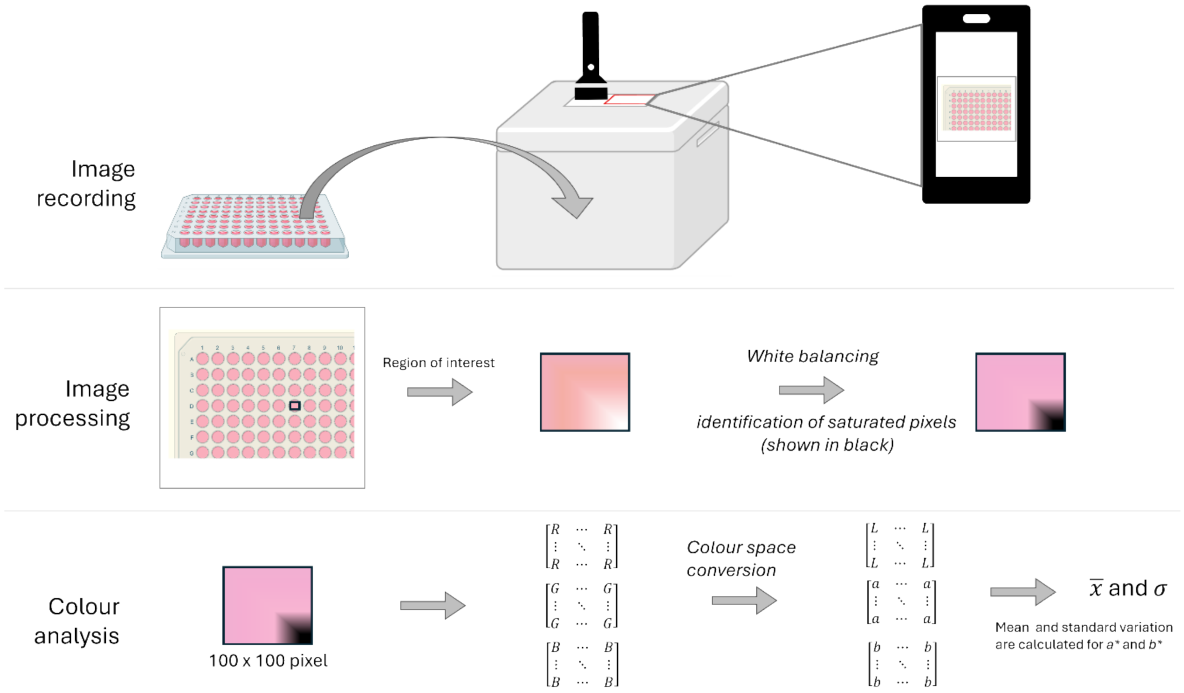

Figure S2. Schematic representation of the CIELAB image analysis. Images are recorded in a white box to ensure constant lightning conditions (first row), then the images are processed or white balancing and identification of staturated pixels, shown in black (second row), and finally the average a* and b* values for each region of interest is determined (third row). For details, please see the experimental section of the main manuscript.

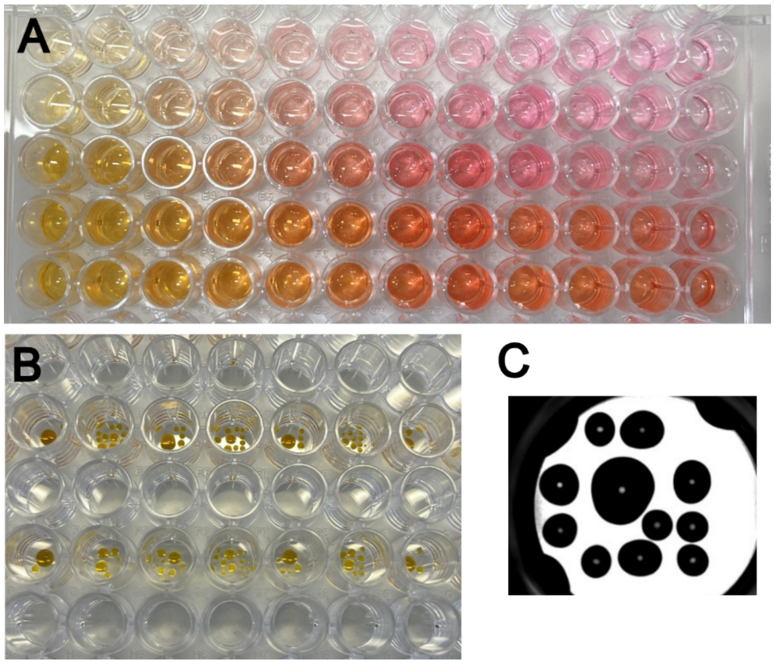

Figure S3. Cellulase activity assay performed in microwell plate. A) Image of wells after reaction using soluble ReC. Corresponding data is presented in Figure 2A in the main

manuscript. Rows: increasing cellulase concentration from left to right. Columns: Increasing ReC concentration from top to bottom. B) Image of wells direct after spotting ReC (top row: 5 mM, bottom row: 2 mM; 5×5 microarray). C): Image taken via in-line camera after spotting 2.5 µL of 2 mM ReC in a 5×5 microarray.

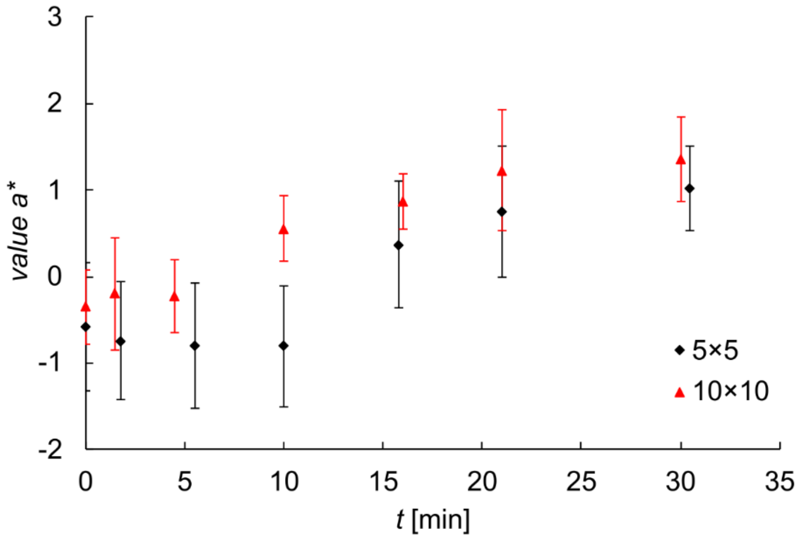

Figure S4. Evolution of value *a** during reaction of spotted ReC applying 5 mg/mL cellulase after spotting of 0.6 µL (2 mM) in a 5×5 and 10×10 microarray. In acetate buffer containing 5 vol% DMSO and 0.1 vol% Triton X-100.

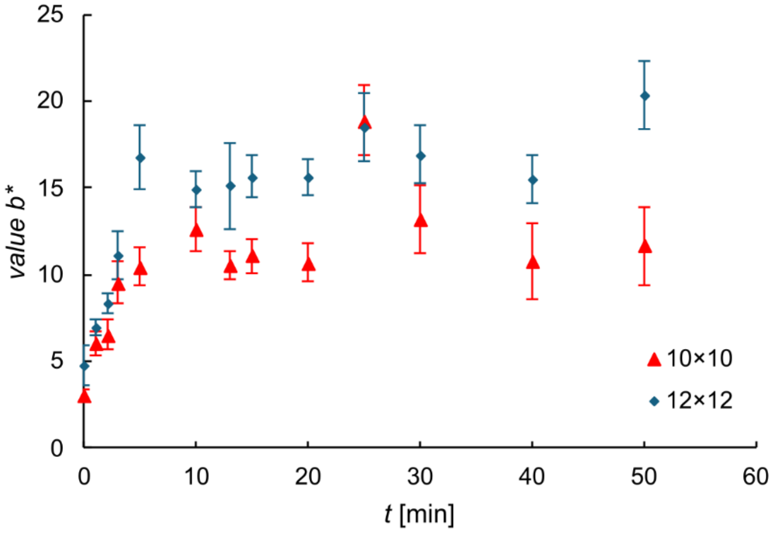

Figure S5. Comparison of solubilization of ReC after spotting a 10×10 and 12×12 microarray based on evaluation of value *b**. Spotting conditions: 2 mM ReC in DMSO, 0.6 µL in total. Sample contained 5 mg/mL in acetate buffer, 7.5 vol% DMSO and 0.1 vol% Triton X-100.
